# Obesity mediates the opposite association of education and diabetes in Chinese men and women: Results from the REACTION study

**DOI:** 10.1111/1753-0407.13325

**Published:** 2022-10-11

**Authors:** Yuanyue Zhu, Chunyan Hu, Lin Lin, Shuangyuan Wang, Hong Lin, Yanan Huo, Qin Wan, Yingfen Qin, Ruying Hu, Lixin Shi, Qing Su, Xuefeng Yu, Li Yan, Guijun Qin, Xulei Tang, Gang Chen, Min Xu, Yu Xu, Tiange Wang, Zhiyun Zhao, Zhengnan Gao, Guixia Wang, Feixia Shen, Zuojie Luo, Li Chen, Qiang Li, Zhen Ye, Yinfei Zhang, Chao Liu, Youmin Wang, Shengli Wu, Tao Yang, Huacong Deng, Lulu Chen, Tianshu Zeng, Jiajun Zhao, Yiming Mu, Weiqing Wang, Guang Ning, Yufang Bi, Yuhong Chen, Jieli Lu

**Affiliations:** ^1^ Department of Endocrine and Metabolic Diseases, Shanghai Institute of Endocrine and Metabolic Diseases, Ruijin Hospital Shanghai Jiao Tong University School of Medicine Shanghai China; ^2^ Shanghai National Clinical Research Center for Metabolic Diseases, Key Laboratory for Endocrine and Metabolic Diseases of the National Health Commission of the PR China, Shanghai Key Laboratory for Endocrine Tumors, State Key Laboratory of Medical Genomics, Ruijin Hospital Shanghai Jiao Tong University School of Medicine Shanghai China; ^3^ Jiangxi Provincial People's Hospital Affiliated to Nanchang University Nanchang China; ^4^ The Affiliated Hospital of Luzhou Medical College Luzhou China; ^5^ The First Affiliated Hospital of Guangxi Medical University Nanning China; ^6^ Zhejiang Provincial Center for Disease Control and Prevention Hangzhou China; ^7^ Affiliated Hospital of Guiyang Medical University Guiyang China; ^8^ Xinhua Hospital Affiliated to Shanghai Jiaotong University School of Medicine Shanghai China; ^9^ Tongji Hospital, Tongji Medical College, Huazhong University of Science and Technology Wuhan China; ^10^ Sun Yat‐sen Memorial Hospital, Sun Yat‐sen University Guangzhou China; ^11^ The First Affiliated Hospital of Zhengzhou University Zhengzhou China; ^12^ The First Hospital of Lanzhou University Lanzhou China; ^13^ Fujian Provincial Hospital, Fujian Medical University Fuzhou China; ^14^ Dalian Municipal Central Hospital Affiliated of Dalian Medical University Dalian China; ^15^ The First Hospital of Jilin University Changchun China; ^16^ The First Affiliated Hospital of Wenzhou Medical University Wenzhou China; ^17^ Qilu Hospital of Shandong University Jinan China; ^18^ The Second Affiliated Hospital of Harbin Medical University Harbin China; ^19^ Central Hospital of Shanghai Jiading District Shanghai China; ^20^ Jiangsu Province Hospital on Integration of Chinese and Western Medicine Nanjing China; ^21^ The First Affiliated Hospital of Anhui Medical University Hefei China; ^22^ Karamay Municipal People's Hospital Xinjiang China; ^23^ The First Affiliated Hospital of Nanjing Medical University Nanjing China; ^24^ The First Affiliated Hospital of Chongqing Medical University Chongqing China; ^25^ Union Hospital Tongji Medical College, Huazhong University of Science and Technology Wuhan China; ^26^ Shandong Provincial Hospital affiliated to Shandong University Jinan China; ^27^ Chinese People's Liberation Army General Hospital Beijing China

**Keywords:** diabetes, education, gender‐dependent, mediation, obesity, 教育, 糖尿病, 性别依赖, 中介, 肥胖

## Abstract

**Background:**

Evidence regarding the impact of education on diabetes risk is scarce in developing countries. We aimed to explore the association between education and diabetes within a large population in China and to identify the possible mediators between them.

**Methods:**

Information on educational level and lifestyle factors was collected through questionnaires. Diabetes was diagnosed from self‐report and biochemical measurements. A structural equation model was constructed to quantify the mediation effect of each mediator.

**Results:**

Compared with their least educated counterparts, men with college education had a higher risk of diabetes (odds ratio [OR] 1.19; 95% confidence interval [CI], 1.12–1.27), while college‐educated women were less likely to have diabetes (OR 0.77; 95% CI, 0.73–0.82). Obesity was the strongest mediator in both genders (proportion of mediation: 11.6% in men and 23.9% in women), and its association with education was positive in men (β[SE] 0.0387 [0.0037]) and negative in women (β[SE] −0.0824 [0.0030]). Taken together, all behavioral factors explained 12.4% of the excess risk of diabetes in men and 33.3% in women.

**Conclusions:**

In a general Chinese population, the association between education level and diabetes was positive in men but negative in women. Obesity was the major mediator underlying the education disparities of diabetes risk, with a stronger mediation effect among women.

## Graphical abstract

**Highlights**

- The association between education level and diabetes in Chinese adults is positive in men but negative in women.
- Obesity is the major mediator underlying the gender disparity in the association between education and diabetes.
- Combating obesity may help address the burden of diabetes across education levels in both genders.

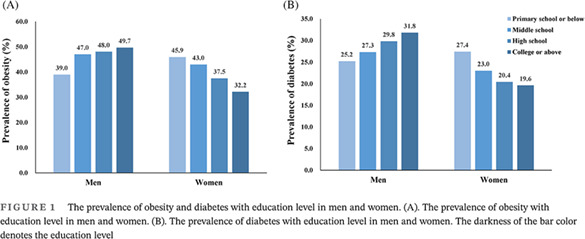

## 摘要

**目的:**

在发展中国家，关于教育对糖尿病风险的影响的证据很少。我们的目的是在中国的一个大人群样本中探索教育和糖尿病之间的联系，并确定可能的中介因素。

**材料和方法:**

通过问卷调查，收集有关文化程度和生活方式因素的信息。糖尿病的诊断是通过自我报告和生化测量进行的。我们构建了结构方程模型量化了各变量的中介效应。

**结果:**

与受教育程度最低的男性相比，受过大学教育的男性患糖尿病风险较高(优势比OR:1.19, 95%可信区间(95%CI):1.12~1.27)，而受过大学教育的女性患糖尿病风险较低(OR:0.77, 95%CI:0.73~0.82)。肥胖在两性中均是最强的中介因素(中介比例:男性11.6%，女性23.9%)，肥胖与教育呈正相关(β[SE]0.0387[0.0037])，与女性中负相关(β[SE]‐0.0824[0.0030])。所有行为因素加在一起，可以解释12.4%的男性和33.3%的女性患糖尿病的额外风险。

**结论:**

在中国普通人群中，男性受教育程度与糖尿病之间存在正相关，而女性则为负相关。肥胖是糖尿病风险教育差异的主要中介因素，在女性中的中介作用更强。

## 1 INTRODUCTION

China is the biggest developing country in the world and is currently undergoing a drastic nutrition transtion. The past decades have witnessed an alarming increase in diabetes prevalence, from 5.5% in 2001^1^ to 12.8% in 2017,^2^ which is much faster than that in developed countries. To halt the striking rising trend of diabetes, it is essential to identify those at high risk and implement target population‐level strategies.

Low education level is a well‐established risk factor of diabetes in developed countries.^3^, ^4^, ^5^ However, in developing countries such as China, the association varied with time and region.^6^, ^7^, ^8^ Therefore, it is crucial to renew the related knowledge in a nationally representative cohort of the Chinese population.

Previous studies investigating the education–diabetes association have already identified several possible mediators.^9^, ^10^, ^11^ Unhealthy behaviors like obesity, smoking, and depressive symptoms were shown to play a role in this association.^9^, ^12^, ^13^ However, few studies assessed the specific mediating effect of each mediator separately. The knowledge gap added the difficulties in understanding the educational disparities of diabetes risk. Indeed, in most previous studies, the mediation effect of a certain variable was discovered by the difference in the estimates between adjusted and unadjusted models.^14^, ^15^, ^16^ Admittedly, such a design has methodological issues as the inclusion of a single variable might alter the convergence of the whole statistical model, and subsequently affect the estimates of each mediation effect in an uncontrollable way. Therefore, an advanced statistical method tailored for mediation analysis is warranted for evaluating the path‐specific effects of interest.^17^

Furthermore, evidence for sex differences in the education–diabetes association has been indicated in several studies, where the link between education and diabetes seemed stronger among women than men,^18^, ^19^ or even in the opposite direction.^3^, ^20^ Therefore, it might be necessary to separate the discussion by gender. Hence, in the current study, we aimed to examine the relationship between education level and diabetes in both genders. We also tried to search for possible mediators, and individually assess the specific mediation effect of each mediator. Obesity, smoking history, unhealthy diet and activity status, non‐ideal sleep time, and depression are all well‐recognized behavioral risk factors of diabetes in previous literature; more importantly, they are all modifiable factors, which have the potential to be corrected. Given this, these variables were chosen to be the potential mediators in the current mediation study.

## 2 METHODS

### 2.1 Study population

The REACTION study is a multicenter, nationwide, population‐based study conducted in Chinese individuals aged 40 years or older.^21^ Briefly, 259 657 participants were recruited from 25 communities across mainland China, with 253 490 individuals providing information on education level. Among them, 8609 participants were further excluded because of missing information on both diabetes diagnosis and biomedical measurement. Finally, a total of 244 881 individuals were included in the analysis. The study was approved by the Committee on Human Research at Ruijin Hospital, and written informed consent was obtained from each participant.

### 2.2 Data collection and clinical evaluation

Information on medical history, education level, residence, occupation, lifestyle factors (including smoking behavior, physical activity, and dietary patterns), and family history of diabetes was collected via in‐person interviews with a standard questionnaire. In the current study, participants were interviewed about their highest attained education. In response of the question, the participants were categorized into four groups (i.e., primary school or below, middle school, high school, and college or above). A validated questionnaire (Patient Health Questionnaire 9, PHQ‐9) was also administered to assess mental health.^22^ Participants underwent measurements of height and weight using a standard protocol with light clothes and no shoes. Body mass index (BMI) was calculated as body weight in kilograms divided by body height in meters squared (kg/m^2^).

After an overnight fast of at least 10 h, blood samples were drawn for biochemical tests. Plasma was obtained at 0 and 2 h for the measurement of fasting glucose and 2 h postload glucose. Plasma glucose concentrations were evaluated at local hospitals using the glucose oxidase or hexokinase method within 2 h after blood sample collection. The Hemoglobin Capillary Collection System (Bio‐Rad Laboratories, Hercules, California) was used to collect finger capillary whole blood and shipped at 2–6°C to the central laboratory of the study to measure the level of glycosylated hemoglobin (HbA1c).

### 2.3 Definition of exposure, outcome, and covariates

#### 2.3.1 Education levels

Education level was self‐reported and categorized into four groups, including primary education or below, middle school, high school, and college or above. In the current mediation study, education level was further classified into two categories based on receiving high school education or not. In China, primary and middle school education is compulsory; therefore, participants who completed high school were considered relatively well educated.^23^ This classification was also in accordance with that in our previous study.^24^

#### 2.3.2 Diabetes

According to the American Diabetes Association (ADA) 2010 criteria,^25^ diabetes was defined as (1) a self‐reported previous diagnosis by health‐care professionals, (2) fasting plasma glucose level of 126 mg/dl (7.0 mmol/L) or higher, (3) 2‐h plasma glucose level of 200 mg/dl (11.1 mmol/L) or higher, or (4) HbA1c concentration of 6.5% or higher.

#### 2.3.3 Covariates

Behavioral factors included obesity, smoking, physical activity, dietary patterns, and depression. For obesity, the cutoff point for Asian populations was used (BMI ≥ 25 kg/m^2^).^26^ For sleep duration, a sleep time of 6–8 h was considered ideal, as is verified by our previous study.^27^ Smoking status was binarized as ever/never smoker because diabetes risk changes remarkably once smoked.^28^ Healthy physical activity was defined according to the 2008 Physical Activity Guidelines for Americans. Participants who engaged in moderate‐intensity exercise for ≥150 min/week or vigorous‐intensity exercise for ≥75 min/week were considered physically active.^29^ Healthy dietary patterns were defined as a dietary score of 4.^30^ For depressive symptoms, each of the nine items of the PHQ‐9 is scored as 0, 1, 2, or 3, and the total score was summed; a PHQ‐9 score of 5–27 represented overt depression.^22^

Other covariates included in the analysis were age, economic‐geographic residence and family history of diabetes. Economic‐geographic residences of China were categorized into four groups according to the per capita disposable income of households from National Bureau of Statistics of China as Eastern, Northeastern, Central, and Western, which reflected different levels of economic development in China.^31^ Age was defined as the age of the participants at recruitment. Family history of diabetes was defined as diabetes diagnosis among first‐degree relatives (i.e., direct blood relatives).

### 2.4 Statistical analysis

The total effect of education on diabetes was estimated with multivariable logistic regression; odds ratios (ORs) and 95% confidence intervals (95% CIs) of education level (four categories) for diabetes are presented for men and women separately in Table 2. Model 1 was the crude model, model 2 was further adjusted for age, economic‐geographic residence, and family history of diabetes. In model 3, obesity (yes/no), ever smoker (yes/no), healthy diet (yes/no), ideal sleep time (yes/no), healthy physical activity (yes/no), and depression (yes/no) were also adjusted.

For the behavioral factors of interest, multivariable logistic regression was used to estimate their independent effect on diabetes one by one, separately in men and women. Age, education level, economic‐geographic residence, and family history of diabetes were adjusted for the analysis of each behavioral factor. The variables signifiantly associated with diabetes were included in a mediation model, and the mediation effect was further quantified. In this study, mediation analysis was conducted with a structural equation model (SEM), which is a recommended approach for mediation analysis with multiple mediators.^32^, ^33^ SEMs are usually elaborated by path diagrams, with nodes representing the variables and arrows representing the relationship between them.^33^ A schematic diagram is shown in Supplement Figure S2. In the current model, the pathway between education and mediators was denoted as path α, the one between mediators and diabetes as path β, and the overall association of education with diabetes was marked as path c. Age, economic‐geographic residence, and family history of diabetes were used as controlling variables. The indirect effect was obtained by multiplying the coefficients of path α and β. The proportion of mediation for each mediator was calculated by dividing the total effect into indirect effect. SAS version 9.4 (SAS Institute) was used to conduct multivariate logistic regression analyses, and R version 4.0.3 (R Foundation for Statistical Computing) was used to construct an SEM for analyzing the complexity of associations between mediators and outcome using the “Lavvan” package.^34^

## 3 RESULTS

**FIGURE 1:**
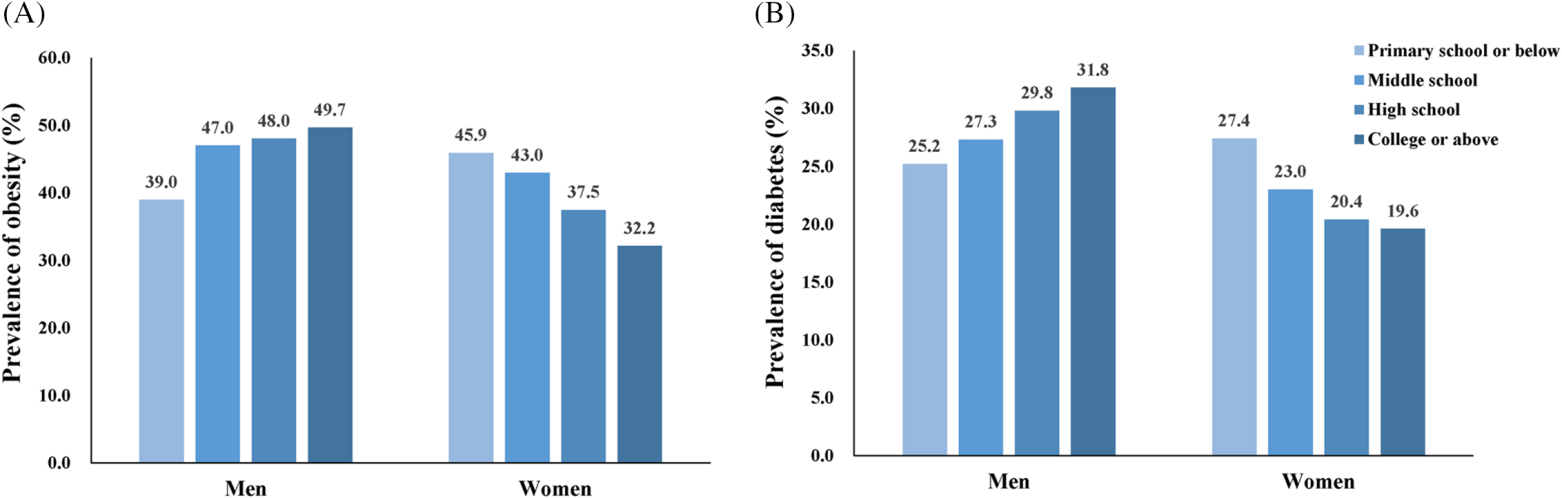
The prevalence of obesity and diabetes with education level in men and women. (A). The prevalence of obesity with education level in men and women. (B). The prevalence of diabetes with education level in men and women. The darkness of the bar color denotes the education level

The distribution of behavioral factors by education level is shown in Table 1 (education level of four categories) and Supplement Table S1 (education level of two categories: high school education or not). Generally, participants with a higher education level tended to adopt an more ideal lifestyle compared with their disadvantaged counterparts: They were more likely to have a healthier diet, sleep time, and physical activity and less likely to smoke. However, the prevalence of both obesity and diabetes changed with education level in the opposite direction by gender. Compared with those with the lowest education level, the prevalence of obesity was 49.6% versus 39.0% in men, and 32.1% versus 45.9% in women with the highest education level, respectively (Figure 1A). Similarly, Figure 1B shows that the prevalence of diabetes rises with increasing education level in a graded manner among men (25.2% for primary school or below and 31.8% for college or above), while in women it decreases as education level rises (27.4% for primary school or below and 19.6% for college or above).

**TABLE 1:** Baseline characteristics according to education level in men and women

	Men				Women			
	Primary school or below	Middle school	High school	College or above	Primary school or below	Middle school	High school	College or above
Number, *n* (%)	19 009	30 117	22 257	13 106	54 860	51 702	41 301	12 529
Age (years)	62.24 ± 9.75	57.31 ± 9.29	56.85 ± 9.49	58.55 ± 10.84	60.79 (9.64)	55.33 (8.60)	54.36 (8.00)	54.61 (9.77)
Body mass index (kg/m^2^)	24.21 ± 3.62	24.86 ± 3.48	24.94 ± 3.51	25.09 ± 3.37	24.86 (3.74)	24.71 (3.64)	24.21 (3.51)	23.84 (3.36)
Obesity, *n* (%)	7291 (39.0)	13 919 (47.0)	10 500 (48.0)	6370 (49.6)	24 850 (45.9)	21 855 (43.0)	15 222 (37.5)	3934 (32.1)
Sleep time (h)	2.59 ± 0.54	2.49 ± 0.55	2.43 ± 0.56	2.40 ± 0.55	2.53 (0.56)	2.41 (0.56)	2.36 (0.55)	2.34 (0.55)
Ideal sleep time (%)	5920 (35.8)	11 973 (45.3)	10 161 (50.3)	6590 (54.2)	19 360 (40.2)	24 166 (52.1)	21 648 (56.6)	6828 (58.6)
Ever smoker, *n* (%)	7627 (40.1)	12 798 (42.5)	8366 (37.6)	3577 (27.3)	840 (1.5)	752 (1.5)	516 (1.2)	94 (0.8)
Healthy diet, *n* (%)	7882 (54.3)	14 368 (57.9)	11 945 (62.8)	7486 (65.5)	22 257 (52.6)	25 828 (59.2)	23 716 (65.6)	7319 (66.5)
Healthy physical activity, *n* (%)	1542 (8.5)	3589 (12.3)	3468 (16.0)	2775 (21.7)	4354 (8.3)	6695 (13.3)	6325 (15.7)	2192 (18.0)
Depression, *n* (%)	498 (2.9)	837 (3.1)	736 (3.6)	523 (4.2)	2360 (4.7)	2282 (4.8)	2200 (5.7)	832 (7.0)
Diabetes, *n* (%)	4782 (25.2)	8208 (27.3)	6625 (29.8)	4167 (31.8)	15 033 (27.4)	11 880 (23.0)	8424 (20.4)	2458 (19.6)
Family history of diabetes, *n* (%)	965 (5.3)	3202 (11.0)	3191 (14.8)	2107 (16.5)	3364 (6.4)	7022 (14.0)	8091 (20.1)	2807 (23.0)
Economic‐geographic residence (%)								
Eastern	10 766 (56.6)	17 492 (58.1)	12 884 (57.9)	7141 (54.5)	29 173 (53.2)	27 382 (53.0)	22 961 (55.6)	6529 (52.1)
Northeastern	4715 (24.8)	4759 (15.8)	2904 (13.0)	1348 (10.3)	13 463 (24.5)	6404 (12.4)	3856 (9.3)	1089 (8.7)
Central	3093 (16.3)	6069 (20.2)	4888 (22.0)	3537 (27.0)	10 619 (19.4)	13 207 (25.5)	10 583 (25.6)	3497 (27.9)
Western	435 (2.3)	1797 (6.0)	1581 (7.1)	1080 (8.2)	1605 (2.9)	4709 (9.1)	3901 (9.4)	1414 (11.3)

*Note*: Continuous variables are represented as mean ± SD and categorical variables are shown as case (%).

The association between education level and diabetes is depicted by gender in Table 2. After adjusting for age, economic‐geographic residence, lifestyle factors, and obesity, the association of diabetes and education level remained positive in men and negative in women. Compared with the least‐educated individuals, the ORs (95% CI) of diabetes among men and women with college education were 1.19 (1.12–1.27) and 0.77 (0.73–0.82), respectively. When taking education level as a dichotomous variable (high school education or not), participants with high school education were at a 11% higher risk in men and 16% lower risk in women, which is shown in Supplement Table S2.

**TABLE 2:** Association between education level and diabetes in men and women

Men	Primary school or below	Middle school	High school	College or above
Case (%)	15 033 (27.4)	11 880 (23.0)	8424 (20.4)	2458 (19.6)
Model 1	Ref	1.12 (1.07–1.16)	1.26 (1.21–1.32)	1.39 (1.32–1.46)
Model 2	Ref	1.26 (1.20–1.31)	1.39 (1.33–1.46)	1.40 (1.33–1.47)
Model 3	Ref	1.14 (1.09–1.21)	1.24 (1.17–1.31)	1.19 (1.12–1.27)
**Women**				
Case (%)	4782 (25.2)	8208 (27.3)	6625 (29.8)	4167 (31.8)
Model 1	Ref	0.79 (0.77–0.81)	0.68 (0.66–0.70)	0.65 (0.62–0.68)
Model 2	Ref	1.02 (0.99–1.05)	0.87 (0.85–0.90)	0.77 (0.73–0.81)
Model 3	Ref	0.98 (0.94–1.01)	0.86 (0.83–0.90)	0.77 (0.73–0.82)

*Note*: Model 1: crude model. Model 2: adjusted for age, sex, and economic‐geographic residence. Model 3: further adjusted for obesity (yes/no), ever smoker (yes/no), healthy diet (yes/no), ideal sleep time (yes/no), healthy physical activity (yes/no), and depression (yes/no).

We then examined the effect of each behavioral factor on diabetes in both genders. Supplement Figure S1 summarizes the ORs of the potential factors on diabetes after controlling for age, economic‐geographic residence, family history of diabetes, and education. In men, obesity and being a smoker were significantly related to the risk of diabetes, while in women, significant variables were obesity, healthy diet, healthy physical activity, and ideal sleep time. Therefore, these factors were included in the mediation model as potential mediators. For men, the proportion of mediation was 11.6% for obesity and 0.8% for being an ever smoker (Table 3), whereas among women, the proportion for mediation was 23.9% for obesity, 3.1% for healthy diet, 2.1% for healthy physical activity, and 4.2% for ideal sleep time (Table 3). Overall, behavioral factors mediated 12.4% and 33.3% of the total association between education and diabetes in men and women.

**TABLE 3:** Mediation analysis of the association between education level and diabetes in men and women

	X → M (α path)		M → Y (β path)		Indirect effect (α*β)		Total effect		Mediation (%)
**Men**	β	SE	β	SE	β	SE	β	SE	
Obesity	0.0387	0.0037	0.1102	0.0030	0.0043	0.0004	0.0367	0.0034	11.6%
Ever smoker	−0.0806	0.0035	−0.0037	0.0031	0.0003	0.0003			0.8%
**Women**	β	SE	β	SE	β	SE			
Obesity	−0.0824	0.0030	0.1121	0.0024	−0.0092	0.0004	−0.0387	0.0025	23.9%
Healthy diet	0.0669	0.0029	−0.0179	0.0027	−0.0012	0.0002			3.1%
Healthy physical activity	0.0500	0.0023	−0.0164	0.0035	−0.0008	0.0002			2.1%
Ideal sleep time	0.0783	0.0030	−0.0207	0.0024	−0.0016	0.0002			4.2%

*Note*: Age, economic‐geographic residence, and family history of diabetes were used as controlling variables.

## 4 DISCUSSION

In the present study, we found that the association of diabetes with education level was positive in men but negative in women. Moreover, obesity was the major mediator underlying the observed association. To our knowledge, this is the first mediation analysis in a Chinese population concerning the gender‐specific education–diabetes association. These results bring new perspectives to the prevention of diabetes at the population level.

Compared with other indicators, education is superior in representing socioeconomic levels. Education level can incorporate socioeconomic factors in both earlier and later life and remains relatively stabilized throughout the life course. Furthermore, information on education is less private and thus more easily to obtain and measure.^35^ As a result, education was employed as proxy to represent socioeconomic position in our study, as is the case in much of the proceeding literature.^36^, ^37^

To date, a number of studies have explored the association between educational level and diabetes, with some further discussing the potential mediators. However, the results were conflicting with diverse economic development and ethnicity. For example, in developed countries, the association between educational level and diabetes is unitedly negative^5^, ^38^ while in less developed countries, such as China and India, the results were far more discordant.^39^, ^40^ Even within the Chinese population, the relationship varied with time, region, and overall education level of the study population. In 2006, a study conducted in Nanjing reported that participants with higher education were at higher risk of diabetes after adjustment for gender,^39^ while in a previous Qingdao study, low education was an established risk factor of diabetes.^41^ Another study at approximately the same period using the China Kadoorie Biobank (CKB) population reported no liner association between education and diabetes.^20^ However, in contrast to all these studies, a gender‐specific gradient relationship between education and diabetes was observed in our study, with men with a high level of education and women with a low level of education having a high risk of diabetes. This finding is in line with what was found in Korean^42^ and Swedish populations,^14^ where the ORs of diabetes among men with higher education and women with lower education ranged from 1.64 to 2.3. It is indicated that gender differences in the education–diabetes association are most likely to occur in low‐ or middle‐income countries which are currently undergoing economic and nutritional transformation.^8^, ^43^

The gendered relationship between education and diabetes could be a result of the variation of BMI with education, as Wu et al. put it.^20^ However, no further elucidation was given in that study. Similarly, we also found that obesity is the major mediator in both genders. Given its nonnegligible mediation effect size and the opposite connection with education level (positive in men and negative in women), it might be one possible explanation in the contrasting association between education and diabetes by gender.

Nevertheless, other mediating factors varied in type and quantity by gender. Apart from obesity, smoking for men, physical activity, diet, and sleep time for women also played important mediating roles in the education–diabetes relationship. For obesity alone, the mediation proportion of obesity was 23.9% in women, more than double that (11.6%) in men. One possible explanation is that the association between education and obesity is stronger in women (path α in Table 2). The association between education level and obesity was stronger among women, which was consistent with a study from Thailand.^44^ Taking all the results together, it could be speculated that behavioral factors have a stronger impact on diabetes among women, which is consistent with previous findings.^14^, ^45^ In this case, maintaining a healthy lifestyle might be most useful for the prevention of diabetes among women with low education.

Furthermore, it is noteworthy that even when all the mediators are combined, they can only explain a modest percentage (33.3% for women and 12.4% for men) in the total effect of education on diabetes, which was reported before as well.^46^, ^47^ Thus, it is implied that other potential mediators such as marital status, household income, and insurance type still remained undiscovered, and the direct effect of education is remarkable. Nevertheless, efforts aiming at lifestyle modification should be encouraged in people with low education, particularly women.

The strengths of this study include the large nationally representative sample, the comprehensive inclusion of potential mediators, and the advanced statistical method for mediation analysis. However, there are some limitations to be addressed. Firstly, it was a cross‐sectional study, which limits our interpretation of causality underlying the association between education and diabetes. However, the highest educational level usually remained consistent after early adulthood^48^ and thus less likely led to a reversed causation. Secondly, information on education level, diet, and physical activity was self‐reported, which might result in a reported bias. Thirdly, people aged less than 40 years old were not included in the current study due to our study design. However, as shown in previous studies, the education–diabetes association is more evident among younger individuals,^49^ which means our results might underestimate, but not overestimate, the total effect of education on diabetes.

In conclusion, we found a gender‐specific association between education and diabetes in China, where women with low education and men with high education were at higher risk of diabetes. The mediation effect of obesity might be one possible explanation in this gender difference. Other behavioral factors such as physical activity and sleep time may also play a mediating role, but this is only evident in women. Therefore, mitigating obesity is of great potential to curb the educational disparities of diabetes risk in both genders, and promoting a healthy lifestyle is especially helpful for women.

## DISCLOSURE

None declared.

## Supporting information


**Appendix S1** Supporting Information.
