# Supplementary material for: Obesity mediates the opposite association of education and diabetes in Chinese men and women: Results from the REACTION study
Source: J Diabetes. 2022 Oct 11;14(11):739–48. doi: 10.1111/1753-0407.13325 (PMC9705800; doi:10.1111/1753-0407.13325)
Supplement: Supplementary file 1 — Appendix S1 Supporting Information. [file JDB-14-739-s001.docx]

**Supplementary materials:**

**Supplement Table 1: Baseline characteristics according to high school education (yes/no) in men and women.**

|  | **Men** | | | **Women** | | |
| --- | --- | --- | --- | --- | --- | --- |
|  | Highschool (-) | Highschool (+) | *P* value | Highschool (-) | Highschool (+) | *P* value |
| Number, n (%) | 35363 (41.86) | 49126 (58.14) |  | 53830 (21.98) | 106562 (43.52) |  |
| Age | 59.22 ± 9.77 | 57.48 ± 10.04 | <0.001 | 58.14 ± 9.55 | 54.42 ± 8.44 | <0.001 |
| Body mass index | 24.61 ± 3.55 | 24.99 ± 3.46 | <0.001 | 24.79 ± 3.69 | 24.13 ± 3.48 | <0.001 |
| Obesity | 21210 (43.9) | 16870 (48.6) | <0.001 | 46705 (44.5) | 19156 (36.2) | <0.001 |
| Ideal sleep time, n (%) | 17893 (41.6) | 16751 (51.8) | <0.001 | 43526 (46.0) | 28476 (57.1) | <0.001 |
| Ever smoker, n (%) | 49126 (57.3) | 35363 (42.7) | <0.001 | 106562 (66.2) | 53830 (33.7) | <0.001 |
| Healthy diet, n (%) | 22250 (56.6) | 19431 (63.8) | <0.001 | 48085 (56.0) | 31035 (65.8) | <0.001 |
| Ideal physical activity, n (%) | 5131 (10.9) | 6243 (18.1) | <0.001 | 11049 (10.8) | 8517 (16.2) | <0.001 |
| Depression, n (%) | 1335 (3.0) | 1259 (3.8) | <0.001 | 4642 (4.7) | 3032 (6.0) | <0.001 |
| Diabetes, n (%) | 12990 (26.4) | 10792 (30.5) | <0.001 | 26913 (25.3) | 10882 (20.2) | <0.001 |
| Family history of diabetes, n (%) | 4167 (8.8) | 5298 (15.4) | <0.001 | 10386 (10.1) | 10898 (20.8) | <0.001 |

**Supplement Table 2: Association between high school education and diabetes in men and women.**

|  | **Men** | | **Women** | |
| --- | --- | --- | --- | --- |
|  | High school (-) | High school (+) | High school (-) | High school (+) |
| Case (%) | 12990 (26.4) | 10792 (30.5) | 26913 (25.3) | 10882 (20.2) |
| Model 1 | Ref | 1.22(1.19-1.26) | Ref | 0.75(0.73-0.77) |
| Model 2 | Ref | 1.17(1.13-1.20) | Ref | 0.82(0.80-0.84) |
| Model 3 | Ref | 1.11(1.06-1.15) | Ref | 0.84(0.82-0.87) |

Model 1: crude model;

Model 2: adjusted for age, sex and economic–geographic residence;

Model 3: further adjusted for obesity (yes/no), ever smoker (yes/no), healthy diet (yes/no), ideal sleep time (yes/no), heathy physical activity (yes/no), and depression (yes/no)

**Supplement Figure 1: Association between behavioral factors and diabetes.**

**
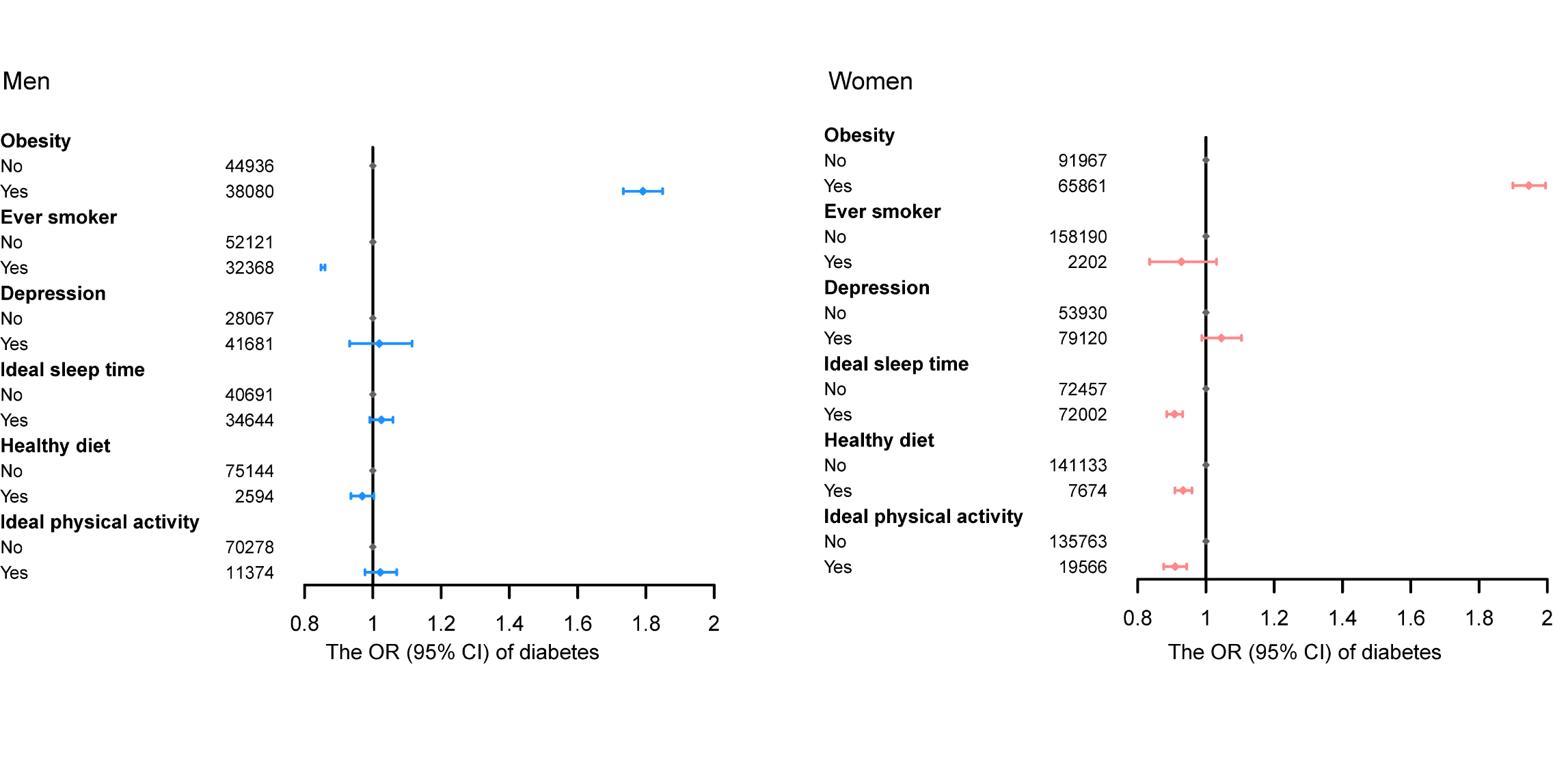
**

The odds ratio of diabetes with behavioral factors in men (A) and women (B) were shown in Figure 1. The ORs were adjusted for the controlling variables (age, family history of diabetes and economic-geographic residence).

**Supplement Figure 2:** **Simple mediation diagram in men and women.**

**
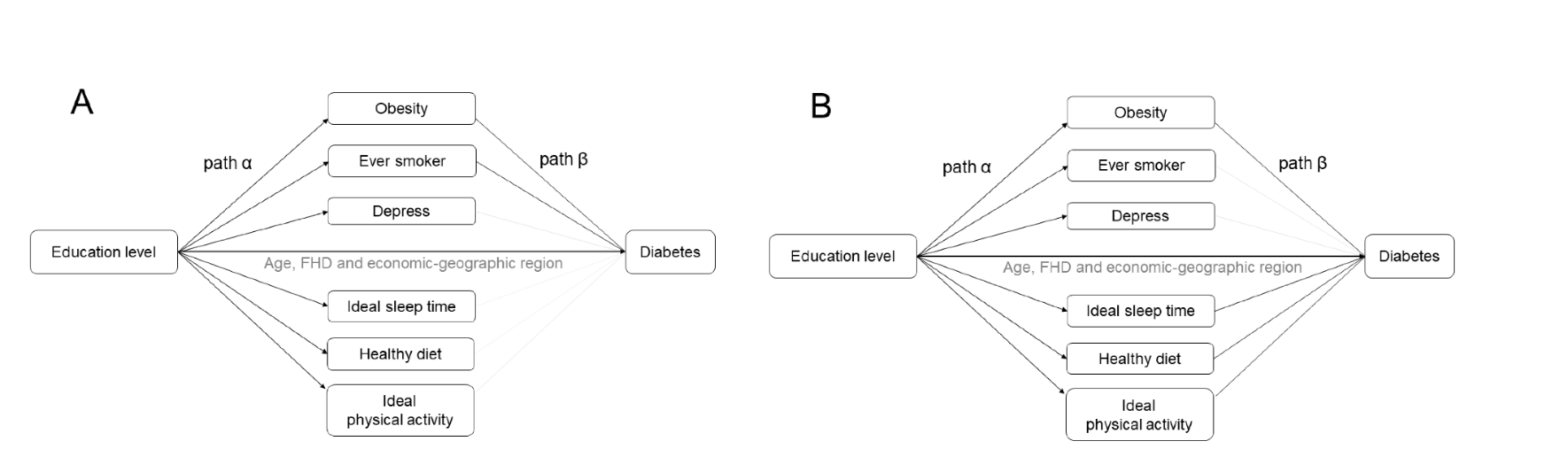
**

Diagram of mediation pathways in men (A) and (B). path α denotes the pathway from exposure (education level) and mediators, path β denotes the pathway from mediators to outcome (diabetes).Age, family history of diabetes (FHD) and economic-geographic residence were used as controlling variables.
